# Real‐World Response and Survival Outcomes and Treatment Patterns in Patients With Extensive Stage Small‐Cell Lung Cancer Receiving Third‐Line Treatment

**DOI:** 10.1002/cnr2.70289

**Published:** 2025-08-12

**Authors:** Jair Bar, Qingqing Xu, Sudeep Karve, Pooja Hingorani, Amin A. Virani, Neal E. Ready

**Affiliations:** ^1^ Jusidman Institute of Oncology, Sheba Medical Center Ramat Gan Israel; ^2^ Gray Faculty of Medical & Health Sciences, Tel Aviv University Tel Aviv‐Yafo Israel; ^3^ AbbVie Inc. North Chicago Illinois USA; ^4^ Duke Cancer Institute, Duke University Durham North Carolina USA

**Keywords:** clinical outcomes, extensive stage, real‐world, SCLC, third‐line, treatment patterns

## Abstract

**Background:**

Patients with small‐cell lung cancer (SCLC) primarily present with extensive stage (ES) disease and poor 5‐year survival. There are few treatment options and limited data on outcomes in third‐line (3L) ES SCLC to quantify unmet medical needs. This chart review evaluated real‐world (RW) clinical outcomes and treatment patterns in these patients.

**Methods:**

This retrospective study using electronic medical records included adult patients with ES SCLC (≥ 2 claims for metastasized lung cancer) who received ≥ 3 lines of therapy (LOT) at Cancer Treatment Centers of America (now City of Hope). Patients with other/multiple primary malignancies, non‐SCLC histology, or treated through a clinical trial were excluded. Clinical outcomes included RW physician‐reported response rates, duration of response (DOR), duration of clinical benefit (DoCB), overall survival (OS), and progression‐free survival (PFS), stratified by platinum sensitivity and Eastern Cooperative Oncology Group performance status (ECOG‐PS). Time‐to‐event outcomes were estimated via Kaplan–Meier. Treatment patterns were assessed, including the sequence of regimens by LOT and first‐line (1L) platinum sensitivity status.

**Results:**

Among 113 eligible patients (median age 58 years), 54% were female, and 46% had a chemotherapy‐free interval after 1L of < 90 days. Tumor shrinkage, complete response, and stable disease were reported in 19%, 1%, and 13% of patients at 3L, respectively. The median DOR, DoCB, OS, and PFS were 2.8, 2.5, 5.8, and 2.4 months in 3L, respectively. Platinum‐resistant versus sensitive patients in 1L had shorter time‐to‐event outcomes. Clinical outcomes were similar regardless of ECOG‐PS status. Platinum‐based chemotherapy rates decreased from 94% and 93% in 1L to 17% and 16% in 3L in platinum‐sensitive and resistant patients, respectively.

**Conclusion:**

These RW findings validate 3L results from clinical trials, demonstrating the limited clinical benefits with current therapies in 3L ES SCLC, and highlighting the urgent need for novel therapies that improve outcomes in this difficult‐to‐treat patient population.

## 1 Introduction

Small‐cell lung cancer (SCLC) is the most aggressive form of lung cancer, constituting 10%–15% of all lung cancer cases, and characterized by a high proliferation rate, rapid tumor doubling time, early onset of widespread metastases, and initial sensitivity to chemotherapy and radiotherapy [1, 2, 3]. Current guidelines suggest the use of a combined approach for staging that incorporates both the tumor, node, metastases, and Veterans Administration staging systems, and categorizes the disease into either limited stage or extensive stage (ES) disease [4, 5]. Given its aggressive nature, patients often (60%–70%) present with ES SCLC, rapidly progress after initial therapy, and have poor overall survival (OS) rates [6, 7, 8].

Platinum‐based doublet with etoposide, or alternatively with irinotecan, has historically been the mainstay of treatment for ES SCLC [9]. After decades of limited clinical progress, the recently introduced immune checkpoint inhibitors including atezolizumab and durvalumab have demonstrated modestly longer survival when combined with chemotherapy, leading to a new standard of care as first‐line (1L) treatment for these patients [10, 11, 12, 13]. The regimens for initial systemic therapy include 4–6 cycles of carboplatin or cisplatin with etoposide and with atezolizumab or durvalumab, followed by maintenance atezolizumab or durvalumab [4].

Patients with ES SCLC frequently relapse not long after the end of chemotherapy treatments or even during the induction phase. Choice of subsequent regimen selection in the second‐line (2L) setting is partly based on the time interval between 1L induction completion and disease progression; patients who progress ≥ 3 months after the end of 1L chemotherapy induction phase are commonly re‐treated with the original regimen, whereas when this interval is shorter than 6 weeks (early relapse), patients are usually treated with topotecan [1, 2]. The recently reported DeLLph‐i304 trial demonstrated superiority of tarlatamab over chemotherapy, supporting its regulatory approval for 2L treatment (Mountzios et al., NEJM 2025). In general, those patients able to receive 2L treatment typically progress at shorter time intervals after 2L initiation with a poor prognosis [14]. Both pembrolizumab and nivolumab were granted accelerated approval by the US Food and Drug Administration for third‐line (3L) SCLC, but approvals were later withdrawn as confirmatory trials did not demonstrate clinical benefit [15]. Currently, no guideline recommendations are available for standard treatment in this setting given the rapid disease progression and poor performance status. There is therefore a great unmet need for novel treatments to extend survival in these patients [2, 14]. Ongoing clinical trials are being conducted to investigate the efficacy of novel agents in the relapsed setting, including lurbinectedin, sacituzumab govitecan, and tarlatamab [16, 17, 18, 19].

There are few clinical trials that have included patients in the 3L setting, and clinical outcome data reported from these trials are limited. A phase 1 study of patients with SCLC treated with doxorubicin combined with lurbinectedin reported a partial response in only one of five patients in the 3L setting, with the remaining four having progressive disease. Median duration of response (DOR) was 2.8 months and median progression‐free survival (PFS) was 1.2 months; this study was limited by its small population of 3L patients evaluable for efficacy (*n* = 5) [20]. The phase 2 TRINITY study of 339 patients treated with the antibody‐drug conjugate rovalpituzumab tesirine in the 3L and beyond setting reported a median DOR of 4.0 months, median OS of 5.6 months, and median PFS of 3.5 months; this study was limited by its single‐arm design and heterogeneity of the patient population [21].

To address evidence gaps in the 3L setting, we designed a retrospective chart review, aiming to determine real‐world physician‐reported response rates, DOR, duration of clinical benefit (DoCB), OS, and PFS as well as treatment patterns in patients with 3L ES SCLC. We have further investigated findings by 1L platinum‐sensitivity status and by 3L Eastern Cooperative Oncology Group performance status (ECOG‐PS).

## 2 Methods

### 2.1 Study Design and Patients

This retrospective, observational real‐world study reviewed electronic medical records (EMR) of patients who received treatment at the Cancer Treatment Centers of America (CTCA, now part of the City of Hope) from July 2012 to June 2022. Eligible patients were aged ≥ 18 years. The metastatic stage of disease was verified by including only patients with ≥ 2 separate claims for metastasized lung cancer using the International Classification of Diseases, Ninth/Tenth Revision, Clinical Modification (ICD‐9/10‐CM) before or on the index date and confirmed in the patients' EMR. Additional inclusion criteria included confirmation of SCLC histological diagnosis (ICD‐O‐3) and ≥ 3 lines of systemic treatment for SCLC (see Supporting Information for prespecified systemic therapies). Systemic treatment was based on the NCCN clinical practice guidelines in Oncology (NCCN Guidelines) for Small‐Cell Lung Cancer v.2.2022, available at the time of study analysis [4]. The index date was the start date of 3L systemic treatment. Patients diagnosed with other/multiple primary malignancies, had histology for non‐SCLC, or who received treatment through a clinical trial were excluded.

### 2.2 Ethics

This was a retrospective study using de‐identified secondary medical health record data of patients; no new data was generated. Therefore, informed consent was not required and not obtained, and institutional review board (IRB) or ethics committee review was not required. The use of secondary de‐identified data is not considered research involving human subjects under Health and Human Services regulations 45 Code of Federal Regulations 46.101(b). Further, research involving the study of existing data is exempt under federal regulations if the information is recorded in such a manner that the patients cannot be identified. The study was conducted in accordance with the Declaration of Helsinki and the International Society for Pharmacoeconomics and Outcomes Research (ISPOR) Good Practices for Outcomes Research.

### 2.3 Outcomes and Assessments

The primary aim of this real‐world study was to evaluate the clinical outcomes of patients with 3L extensive stage SCLC to improve understanding of the unmet need in these patients. Clinical outcomes included physician‐reported response rates, DOR, DoCB, OS, and PFS. Response rates were assessed to the extent that they were documented in medical charts by the health‐care provider and were reported as the percentage of patients having a “complete response,” “tumor shrinkage” (term used in this analysis for a “partial response”), or “stable disease.” At times, there was no documentation of objective response, but a treatment was discontinued or changed to a new line of therapy (LOT); based on the protocol definitions, those situations were deemed “no response,” as it is possible those cases represented symptom or radiological disease progression. Where available, the CTCA team evaluated patient scans for confirmation of response data.

DOR was evaluated among patients with a partial response or complete response and defined as the time from the documented date of tumor shrinkage or complete response to the earliest of the start of the next LOT, disease progression, additional metastatic disease, or death. DoCB was evaluated among patients with a partial response, complete response, or stable disease, and defined as the time from reported tumor shrinkage, complete response, or stable disease to the earliest of the start of the next LOT, disease progression, additional metastatic disease, or death. OS was defined as the time from initiation of a given LOT to death (due to any cause) or end of follow‐up [22]. PFS was defined as the time from initiation of a given LOT to the earliest date of documented progression or death (due to any cause) before the start of a next LOT. Patients without a progression event or death date before the start of a next LOT were censored at the date of treatment discontinuation of that LOT. Clinical outcomes were stratified by patients who were platinum‐sensitive versus platinum‐resistant by time of progression on 1L, and by patients with an ECOG‐PS score of 0–1 versus 2 at 3L, as subgroup analyses. Patients were defined as platinum‐sensitive and platinum‐resistant if they progressed ≥ 3 months or < 3 months after 1L chemotherapy induction discontinuation, respectively.

The sequence of regimens by LOT was evaluated to assess treatment patterns. A Sankey diagram was generated to depict treatment sequences for 1, 2, and 3 LOT. The end of 1L treatment was defined as the earliest observance of the start of a new chemotherapy or immunotherapy agent, re‐start of the same agent of the current regimen if there was a gap in treatment of ≥ 45 days, or if progression was noted in the EMR, with the end date equal to the last day of systemic treatment; if the gap was < 45 days, that was considered as the continuation of the current line. Subsequent LOT were identified using similar rules. Patients were then classified as receiving 1, 2, or ≥ 3 LOT. Subgroup analyses comparing the sequence of 2L and 3L regimens by 1L platinum sensitivity status were conducted.

### 2.4 Analytic Approach

Categorical variables of patient demographics/clinical characteristics (including platinum sensitivity at 1L), treatment response, and treatment regimens by LOT were reported as frequencies and percentages, and continuous variables were reported as median (range).

Time‐to‐event outcomes were estimated using the Kaplan–Meier method and reported as median (interquartile range [IQR] or 95% confidence interval [CI]) in months. Patients were censored at the end of follow‐up or the end of data availability. DOR and DoCB were estimated only among patients with documented responses. Outcomes were reported overall and were compared by 3L ECOG‐PS (ECOG‐PS score = 0–1 vs. ECOG‐PS score = 2) and 1L platinum sensitivity (platinum‐sensitive vs. platinum‐resistant) using Chi‐square, Kruskal‐Wallis, or log‐rank tests. No correction was done for multiple comparisons. For the subgroup analyses, differences in response rates were tested using Chi‐square tests for categorical variables. Differences were considered statistically significant if the *p*‐value was < 0.05.

Sankey diagrams were produced to visualize treatment patterns, including the sequence of systemic treatment by LOT and by platinum sensitivity status in 1L.

## 3 Results

### 3.1 Patient Demographics and Baseline Characteristics

A total of 113 patients with stage III‐IV ES SCLC met the inclusion and exclusion criteria and were included in the analysis (Figure S1). Of these patients, the median (IQR) age at diagnosis was 58 (53, 61) years, 54% were female, and 46% had a chemotherapy‐free interval after 1L of < 90 days. Most were White (87%) and non‐Hispanic (87%) and presented with ES disease at 1L (88%); (Table 1).

**TABLE 1:** Patient baseline demographics and clinical characteristics.

Characteristic	*n* (%) (*N* = 113)
Age (years), median (range)	58 (53–61)
Sex	
Female	61 (54)
Male	52 (46)
Race	
White	98 (87)
Black	9 (8)
Other	1 (1)
Undisclosed	5 (4)
Ethnicity	
Hispanic	2 (2)
Non‐Hispanic	98 (87)
Undisclosed	13 (12)
Smoking history	
Current	31 (27)
Former	55 (49)
Never	3 (3)
Undisclosed	24 (21)
Stage at 1L	
Limited stage	13 (12)
Extensive stage	100 (88)
Platinum sensitivity in 1L	
CTFI ≥ 90 days	45 (40)
CTFI < 90 days	52 (46)
Refractory	16 (14)

Abbreviations: 1L, first‐line; CTFI, chemotherapy‐free interval.

### 3.2 Clinical Outcomes

**FIGURE 1:**
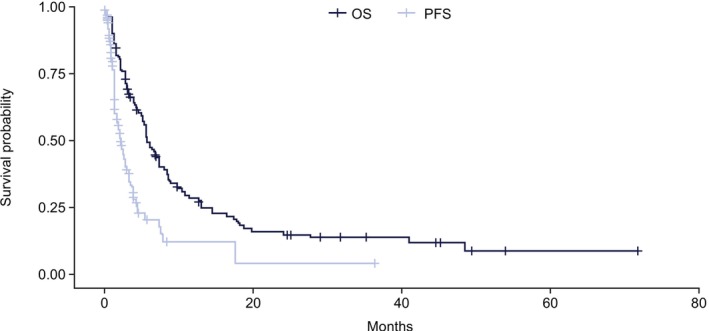
Kaplan–Meier curves of OS and PFS outcomes in patients with 3L extensive stage SCLC. 3L, third‐line; OS, overall survival; PSF, progression‐free survival; SCLC, small‐cell lung cancer.

Of 113 total patients, 19% (*n* = 21) had physician‐reported tumor shrinkage, 1% (*n* = 1) had a complete response, and 13% (*n* = 15) had stable disease at 3L; the remaining 67% (*n* = 76) had no response at 3L. When evaluating responses during earlier LOT among 3L patients, 75% and 27% had tumor shrinkage, 4% and 16% had stable disease, 5% and 3% had a complete response, and 16% and 55% had no response in 1L and 2L, respectively (Table S1). The median (IQR) DOR and DoCB were 2.8 (1.9–3.1) and 2.5 (1.8–3.3) months in 3L, respectively. The median (95% CI) OS and PFS were 5.8 (5.2, 8.1) and 2.4 (1.5, 3.5) months in 3L, respectively (Table 2; Figure 1).

**TABLE 2:** Clinical outcomes in 3L extensive stage SCLC.

Outcome	*N* patients	Median (Q1–Q3) months
DOR^a^	19	2.8 (1.9–3.1)
DoCB^a^, ^b^	29	2.5 (1.8–3.3)

	*N* events	Median (95% CI) months
OS	90	5.8 (5.2–8.1)
PFS	65	2.4 (1.5–3.5)

Abbreviations: 3L, third‐line; CI, confidence interval; DoCB, duration of clinical benefit; DOR, duration of response; OS, overall survival; PFS, progression‐free survival; Q, quartile; SCLC, small‐cell lung cancer.

^a^ Estimated among patients with observed responses.

^b^ Time from reported tumor shrinkage, complete response, or stable disease to progression/no response or next line of therapy.

Subgroup analysis of patients who were platinum‐sensitive (46%; *n* = 52/113) versus platinum‐resistant (40%; *n* = 45/113) in the 1L setting found that patients in the latter group had shorter DOR and DoCB across 1L and 2L of therapy; median DOR was 5.6 versus 2.8 months in 1L (*p* < 0.0001) and 4.2 versus 2.5 months in 2L (*p* = 0.08), and DoCB was 5.6 versus 2.8 months in 1L (*p* < 0.0001) and 4.3 versus 2.6 months in 2L (*p* = 0.008) for platinum‐sensitive versus platinum‐resistant patients, respectively. Similar trends were observed for OS in 1L and PFS in 1L and 2L, with a 1L median OS of 24.5 versus 14.9 months (*p* < 0.0001) and PFS not estimable versus 4.5, and 4.2 versus 2.1 months in 1L (*p* < 0.0001) and 2L (*p* = 0.005) for platinum‐sensitive versus platinum‐resistant patients, respectively (Table 3).

**TABLE 3:** Outcomes by 1L platinum sensitivity and LOT.

Outcome by LOT	Sensitive^a^ in 1L (*N* = 52)	Resistant^b^ in 1L (*N* = 45)	*p value* ^c^
1L, median (Q1–Q3) months			
DOR	5.6 (4.0–7.4)	2.8 (2–3.1)	< 0.0001
DoCB	5.6 (4.0–8.0)	2.8 (2.1–3.1)	< 0.0001
1L, median (95% CI) months			
OS	24.5 (21.9, 36.2)	14.9 (11.9, 17.6)	< 0.0001
PFS	NE	4.5 (4.3, NE)	< 0.0001
2L, median (Q1–Q3) months			
DOR	4.2 (2.9–4.6)	2.5 (2.3–3.3)	0.08
DoCB	4.3 (3.1–5.7)	2.6 (2.1–3.3)	0.008
2L, median (95% CI) months			
PFS	4.2 (2.8, NE)	2.1 (1.5, 3.9)	0.005

Abbreviations: 1L, first‐line; 2L, second‐line; CI, confidence interval; DoCB, duration of clinical benefit; DOR, duration of response; LOT, line of therapy; NE, not estimable; OS, overall survival; PFS, progression‐free survival; Q, quartile.

^a^ Initiating 2L ≥ 3 months after 1L discontinuation.

^b^ Initiating 2L < 3 months after 1L discontinuation.

^c^ Kruskal‐Wallis test.

At 3L treatment initiation, 66% (*n* = 73) of patients had an ECOG‐PS score of 0 or 1 and 33% (*n* = 37) had an ECOG‐PS score of 2. Remaining patients had an ECOG‐PS score of 3 or 4 (2%, *n* = 2), and an ECOG‐PS score was not recorded for one patient. Subgroup analysis of outcomes by ECOG‐PS score in 3L (ECOG‐PS score = 0–1 vs. ECOG‐PS score = 2) found that response rates and time‐to‐event outcomes were not statistically different regardless of ECOG‐PS status. Regarding response rates, 21% (*n* = 15) versus 16% (*n* = 6) had tumor shrinkage, 1% (*n* = 1) versus 0% had a complete response, 12% (*n* = 9) versus 16% (*n* = 6) had stable disease, and 66% (*n* = 48) versus 68% (*n* = 25) had no response for patients with an ECOG‐PS score of 0–1 versus 2, respectively (*p* = 0.79). Regarding time‐to‐event outcomes, median DOR was 2.8 versus 2.9 months (*p* = 0.74), DoCB was 2.1 versus 2.7 months (*p* = 0.69), OS was 6.2 versus 5.1 months (*p* = 0.1), and PFS was 2.4 versus 2.4 months (*p* = 0.78) for patients with an ECOG‐PS score of 0–1 versus 2, respectively (Table 4).

**TABLE 4:** Outcomes for patients with ECOG‐PS score 0–1 versus ECOG‐PS score = 2 at 3L treatment.

Outcome	ECOG‐PS score = 0–1 *N* = 73	ECOG‐PS score = 2 *N* = 37	*p value* ^b^
	*n* (%)	*n* (%)	
Response rate			0.79
No response^a^	48 (66)	25 (68)	
Tumor shrinkage	15 (21)	6 (16)	
Complete response	1 (1)	—	
Stable disease	9 (12)	6 (16)	

	Median (Q1–Q3) months	Median (Q1–Q3) months	*p value* ^d^
DOR^c^	2.8 (1.9–3.0)	2.9 (1.9–3.3)	0.74
DoCB^c^	2.1 (1.9–3.0)	2.7 (1.4–4.4)	0.69
OS	6.2 (5.6–9.8)	5.1 (3.0–8.5)	0.1
PFS	2.4 (1.5–3.5)	2.4 (1.4–NE)	0.78

Abbreviations: 3L, third‐line; DoCB, duration of clinical benefit; DOR, duration of response; ECOG‐PS, Eastern Cooperative Oncology Group performance status; NE, not estimable; OS, overall survival; PFS, progression‐free survival; Q, quartile.

^a^ 2/2 (100%) of patients with an ECOG‐PS score of 3–4 had no response.

^b^ Chi‐square test.

^c^ Analysis based on patients with observed responses.

^d^ Kruskal‐Wallis test.

### 3.3 Treatment Pattern Outcomes

**FIGURE 2:**
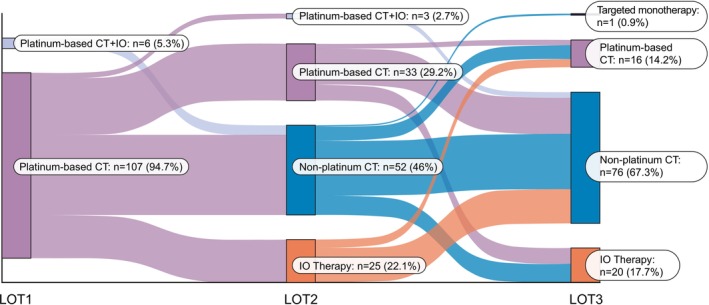
Sankey diagram illustrating treatment sequencing of systemic regimens by LOT. CT, chemotherapy; IO, immunotherapy; LOT, line of therapy.

Among 113 patients, the majority received platinum‐based chemotherapy at 1L (95%), non‐platinum‐based chemotherapy at 2L (46%), and non‐platinum‐based chemotherapy at 3L (67%) (Figure 2). Specifically, the most commonly used regimens at 2L were topotecan (23%), nivolumab (18%), and carboplatin plus irinotecan (11%); and in 3L were irinotecan (20%), docetaxel (15%), and nivolumab (13%).

**FIGURE 3:**
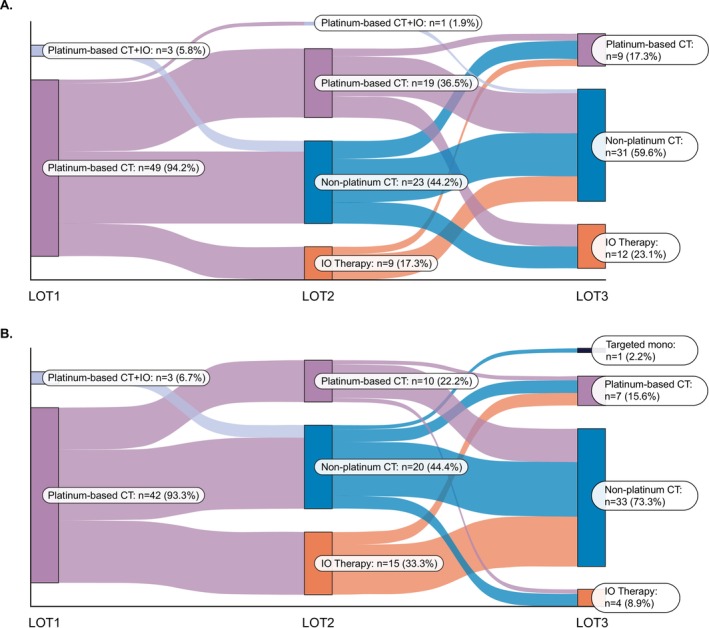
Sankey diagram illustrating treatment sequencing of systemic regimens by LOT, stratified by 1L (A) platinum‐sensitive and (B) platinum‐resistant patients. 1L, first‐line; CT, chemotherapy; IO, immunotherapy; LOT, line of therapy; mono, monotherapy.

The proportion of patients receiving platinum‐based chemotherapy decreased from 94% and 93% in 1L to 17% and 16% in 3L in platinum‐sensitive and resistant patients, respectively (Table 5; Figure 3). Of the 52 patients who were platinum‐sensitive in 1L, the majority received platinum‐based chemotherapy without immunotherapy (IO) at 1L (94%), non‐platinum‐based chemotherapy at 2L (44%), and non‐platinum‐based chemotherapy at 3L (60%) (Figure 3A). Of the 45 patients who were platinum‐resistant in 1L, the majority received platinum‐based chemotherapy without IO at 1L (93%), non‐platinum‐based chemotherapy at 2L (44%), and non‐platinum‐based chemotherapy at 3L (73%) (Figure 3B).

**TABLE 5:** Treatment patterns by 1L platinum sensitivity and LOT.

Regimen categories	Sensitive^a^ in 1L (*N* = 52)	Resistant^b^ in 1L (*N* = 45)
1L, *n* (%)		
Platinum‐based chemotherapy	49 (94.2)	42 (93.3)
Platinum‐based chemotherapy + ICI	3 (5.8)	3 (6.7)
2L, *n* (%)		
ICI therapy	9 (17.3)	15 (33.3)
Non‐platinum chemotherapy	23 (44.2)	20 (44.4)
Platinum‐based chemotherapy	19 (36.5)	10 (22.2)
Platinum‐based chemotherapy + ICI	1 (1.9)	—
3L, *n* (%)		
ICI therapy	12 (23.1)	4 (8.9)
Non‐platinum chemotherapy	31 (59.6)	33 (73.3)
Platinum‐based chemotherapy	9 (17.3)	7 (15.6)
Targeted	—	1 (2.2)

Abbreviations: 1L, first‐line; 2L, second‐line; 3L, third‐line; ICI, immune checkpoint inhibitors; LOT, line of therapy.

^a^ Initiating 2L ≥ 3 months after 1L discontinuation.

^b^ Initiating 2L < 3 months after 1L discontinuation.

## 4 Discussion

Patients with ES SCLC who have progressed to 3L treatment are known to have poor prognosis, given the accumulating drug resistance leading to limited treatment options. There is a lack of guidance on optimal treatment at 3L [6, 7, 8, 14]. In this analysis, real‐world evidence was used to explore the clinical outcomes and treatment patterns of these patients. Findings were further stratified by platinum sensitivity and ECOG‐PS, which are factors previously identified to impact clinical outcomes in SCLC [23, 24]. Results of this chart review may contribute to filling the gap of limited data on outcomes of patients with ES SCLC who have progressed to 3L and assist in guiding the care of these patients.

Clinical outcomes of our study in the 3L setting, including physician‐reported response rates and DOR, were low and validate observations reported in previous clinical trials and real‐world studies in 3L ES SCLC. For example, physician‐reported response rates (tumor shrinkage 19%, complete response 1%, stable disease 13%) observed in this study are in line with results obtained from other published studies that show tumor shrinkage ranging from 7%–21% [20, 25, 26, 27, 28, 29, 30], stable disease from 0%–58% [20, 25, 26, 27, 28, 29, 30], and complete response from 0%–2% [20, 26, 27, 28, 29, 30], highlighting the limited clinical benefits with current therapies in this population. Furthermore, the median DOR of 2.8 months is within the range of prior studies that have reported a median DOR of 2.6–18 months [20, 21, 26, 28]. To our knowledge, this study is the first to evaluate clinical outcomes such as DOR and DoCB among patients with ES SCLC in the 3L setting. The median OS of 5.8 months and PFS of 2.4 months are consistent with previous studies where median OS was observed as 5.6–5.8 months [21, 25, 26, 27, 31] and median PFS previously observed as 2.0–3.1 months [25, 27, 28, 30]. Together these findings suggest there is an unmet need for novel therapies to improve outcomes in patients with 3L ES SCLC.

Findings from our subgroup analysis showed that clinical outcomes of 1L and 2L therapy were better in patients who were platinum‐sensitive at the end of 1L treatment compared with those who were platinum‐resistant with rapid progression after/on 1L treatment, which aligns with previous studies that have determined this correlation [32, 33]. A similar trend has also previously been reported in the 3L setting where platinum sensitivity was associated with better outcomes [34]. These findings are important to note given that resistance to platinum therapy may impact patients' eligibility for additional treatments and impact sequencing of treatments after 1L and 2L therapies.

The results of our analysis also showed that overall, patients with SCLC who survived to receive 3L treatment are a select group with an exceptionally long 1L OS of 24.5 versus 14.9 months for platinum‐sensitive versus platinum‐resistant patients, respectively. Regarding subgroup analysis of outcomes by ECOG‐PS score at 3L, response rates, DOR, DoCB, OS, and PFS were not statistically different regardless of ECOG‐PS score in this study. This contrasts with other studies in SCLC which demonstrated that patients with better ECOG‐PS scores tend to have better outcomes, even in the 3L setting [29, 34]. A possible reason why such a correlation was not observed in the current study may be due to the smaller sample size evaluable for ECOG‐PS subgroup analysis (*n* = 73). Alternatively, patients with ES who survive to receive 3L of therapy are indeed a highly selected group; only 113 patients with ES disease received 3L out of 895 patients with advanced SCLC in the CTCA database (12.6%). It can be speculated that in this selected group, ECOG‐PS may not be a robust prognostic factor. An additional consideration is the limited reliability of the ECOG‐PS designation in retrospective chart reviews [35].

Treatment patterns observed in this study were aligned with guideline recommendations of initial and subsequent systemic therapies for ES SCLC, whereby most patients receive a platinum‐based chemotherapy at 1L and a non‐platinum‐based chemotherapy at 2L [4]. In the 3L setting for which few drugs have been approved and guidance on preferred therapy options is lacking, our study observed that most patients received a non‐platinum‐based chemotherapy. Observations of treatment patterns by LOT in this study are in line with prior real‐world studies [2, 25, 28, 36, 37].

Subgroup analysis of treatment patterns by 1L platinum sensitivity status found that both platinum‐sensitive and platinum‐resistant groups followed the similar treatment patterns for subsequent LOT with similar rates. Conversely, a prior study demonstrated that a majority of patients who were platinum resistant at 1L were rechallenged with platinum‐based therapy as 2L treatment [37]. Such inconsistencies in subsequent preferred treatment options by 1L platinum sensitivity highlight the need for standardizing systemic therapies by these subgroups.

To our knowledge, this study is among the first to assess DOR among ES SCLC in the 3L setting using real‐world databases in the United States. The CTCA database enabled the evaluation of patient response data based on their scans, which is limited in most other accessible real‐world data sources. However, it is important to note that this study has limitations which are similar to other real‐world studies. This study is subject to general limitations of claims data such as inaccuracies in coding of diagnoses, procedures, or pharmacy claims and other unobserved confounders. Evaluation of response based on retrospective chart review is a major challenge. Response was captured by searching for provider notes and image review when available, but not evaluated as per Response Evaluation Criteria in Solid Tumors criteria. Physicians may have ambiguously documented rationales for treatment continuation or change; for example, one might state, “demonstrates response to therapy, stable disease;” when patient scan imaging was not available, the decision to mark a patient as experiencing stable disease or tumor shrinkage was more difficult. In these cases, the research team discussed the physician documentation and decisions about therapy to make a collective decision. Further, when there was no documentation of objective response, but a treatment was discontinued or was changed to a new LOT, patients in those situations were deemed as having “no response,” as it is possible those cases represented symptom or radiological disease progression. This approach assumes that disease progression is often inferred in the absence of documented response; however, there could have been patients who responded but discontinued treatments due to tolerability reasons. As we could not rule out that some patients responded but discontinued for reasons other than disease progression, the results reported here may be a more conservative estimate of the response rate in this patient population. The utilization of time to next LOT as a surrogate for DOR in this study has been partly validated by prior studies [38], however to a limited extent.

Overall, findings from this study demonstrate limited clinical benefits with current standard of care therapies for patients with 3L ES SCLC, highlighting a need for novel therapies. Ongoing clinical trials investigating the efficacy of novel agents in the relapsed setting include lurbinectedin in the phase III LAGOON trial (NCT05153239) [19], sacituzumab govitecan in the phase II TROPiCS‐03 basket trial (NCT03964727) [17], and ifinatamab deruxtecan in the phase III IDeate‐2 trial (NCT06203210) [39]. Tarlatamab has demonstrated promising results in the phase II DeLLphi‐301 trial (NCT05060016) [16] and has recently proven superior to chemotherapy as 2L in the randomized phase III DeLLphi‐304 trial (NCT05740566) [18][Montzios et al., NEJM 2025]. Factors that may influence decision‐making regarding the sequencing and usage of novel agents are multi‐factorial, including reasons for prior treatment discontinuation (e.g., disease progression, treatment tolerability, or patient preference) and patient ECOG‐PS, comorbidities, and eligibility to receive novel therapies. As patients may have developed resistance during prior treatments, the mechanism of resistance should also be carefully evaluated. Further, patients and their treating physicians should discuss the goals for continuing treatment, whether it is for disease control, symptom management, or prolonging survival. The safety and tolerability profile of novel therapies should be carefully considered when determining whether patients are suitable for continuing treatments. Importantly, it should be a shared decision‐making process by both patients and physicians to balance the potential benefits with risks and the impacts on patients' quality of life, particularly in later LOT.

## 5 Conclusion

Findings from this real‐world study were aligned with the findings from prior real‐world studies and clinical trials in the 3L setting, including short DOR, OS, and PFS, indicating that there are limited clinical benefits with current therapies for patients with 3L ES SCLC. Response rates and time to event outcomes in 3L were not statistically different regardless of ECOG‐PS, while 1L platinum‐resistant relative to platinum‐sensitive patients had shorter DOR, DoCB, and PFS in 1L and 2L. Observed treatment patterns from the current study followed what has been observed in prior studies and were in line with guideline recommendations. Together, these findings highlight that more research is warranted to identify novel therapies that improve response and disease control in patients with ES SCLC who have an option of 3L treatment.

## Author Contributions

**Jair Bar:** methodology (co‐lead); writing – original draft (supporting); validation (equal); writing – review and editing (co‐lead). **Qingqing Xu:** conceptualization (lead); methodology (co‐lead), validation (equal); writing – review and editing (equal). **Sudeep Karve:** conceptualization (co‐lead); funding acquisition; supervision; writing – review and editing (equal). **Pooja Hingorani:** methodology (supporting); validation (equal); writing – review and editing (equal). **Amin A. Virani:** conceptualization (supporting); validation (equal); writing – review and editing (equal). **Neal E. Ready:** methodology (co‐lead); validation (equal); writing – review and editing (co‐lead). All authors participated in interpretation of data and all authors approved the final version of the manuscript for publication.

## Ethics Statement

This was a retrospective study using de‐identified secondary medical health record data of patients; no new data was generated. Therefore, informed consent was not required and not obtained and institutional review board (IRB) or ethics committee review was not required. The use of secondary de‐identified data is not considered research involving human subjects under Health and Human Services regulations 45 Code of Federal Regulations 46.101(b). Further, research involving the study of existing data is exempt under federal regulations if the information is recorded in such a manner that the patients cannot be identified. The study was conducted in accordance with the Declaration of Helsinki and the International Society for Pharmacoeconomics and Outcomes Research (ISPOR) Good Practices for Outcomes Research.

## Conflicts of Interest

Jair Bar serves as an advisory board member for AbbVie, AstraZeneca, Bayer, BMS, Causalis, MSD, Novartis, Roche, and Takeda, and has received research funding from AstraZeneca, ImmuneAI, MSD, and OncoHost. Qingqing Xu, Sudeep Karve, Pooja Hingorani, and Amin A. Virani are full‐time employees of AbbVie and may hold AbbVie stock and/or stock options. Neal E. Ready serves as a paid consultant for AstraZeneca, BMS, G1 therapeutics, Jazz, Merck, Regeneron, Roche, and Sanofi, and as an unbranded speaker for BMS and Jazz.

## Supporting information


Data S1.

## Data Availability

This study is a retrospective, non‐interventional cohort study using secondary real‐world data that were already collected during routine clinical practice; no new or primary data were collected, created or analyzed in this study. AbbVie is committed to responsible data sharing regarding the clinical trials we sponsor. This includes access to anonymized, individual, and trial‐level data (analysis data sets), as well as other information (e.g., protocols, clinical study reports, or analysis plans), as long as the trials are not part of an ongoing or planned regulatory submission. This includes requests for clinical trial data for unlicensed products and indications. These clinical trial data can be requested by any qualified researchers who engage in rigorous, independent, scientific research, and will be provided following review and approval of a research proposal, Statistical Analysis Plan (SAP), and execution of a Data Sharing Agreement (DSA). Data requests can be submitted at any time after approval in the US and Europe and after acceptance of this manuscript for publication. The data will be accessible for 12 months, with possible extensions considered. For more information on the process or to submit a request, visit the following link: https://www.abbvieclinicaltrials.com/hcp/data‐sharing/.

## References

[1] M. Dómine , T. Moran , D. Isla , et al., “SEOM Clinical Guidelines for the Treatment of Small‐Cell Lung Cancer (SCLC) (2019),” Clinical and Translational Oncology 22, no. 2 (2020): 245–255. 32040815 10.1007/s12094-020-02295-w

[2] F. Blackhall , N. Girard , A. Livartowski , et al., “Treatment Patterns and Outcomes Among Patients With Small‐Cell Lung Cancer (SCLC) in Europe: A Retrospective Cohort Study,” BMJ Open 13, no. 2 (2023): e052556. 10.1136/bmjopen-2021-052556 PMC9906168 36746549

[3] American Cancer Society , “Key Statistics for Lung Cancer,” (2023), https://www.cancer.org/cancer/types/lung‐cancer/about/key‐statistics.html.

[4] “Referenced With Permission From the NCCN Clinical Practice Guidelines in Oncology (NCCN Guidelines) for Small Cell Lung Cancer v.2.2022. National Comprehensive Cancer Network,” accessed December 14, 2024, To view the most recent and complete version of the guideline, go online to, NCCN.org. NCCN makes no warranties of any kind whatsoever regarding their content, use or application and disclaims any responsibility for their application or use in any way.

[5] E. Vallières , F. A. Shepherd , J. Crowley , et al., “The IASLC Lung Cancer Staging Project: Proposals Regarding the Relevance of TNM in the Pathologic Staging of Small Cell Lung Cancer in the Forthcoming (Seventh) Edition of the TNM Classification for Lung Cancer,” Journal of Thoracic Oncology 4, no. 9 (2009): 1049–1059. 19652623 10.1097/JTO.0b013e3181b27799

[6] E. Arriola , J. M. Trigo , A. Sánchez‐Gastaldo , et al., “Prognostic Value of Clinical Staging According to TNM in Patients With SCLC: A Real‐World Surveillance Epidemiology and End‐Results Database Analysis,” JTO Clinical and Research Reports 3, no. 1 (2022): 100266. 35024640 10.1016/j.jtocrr.2021.100266 PMC8728577

[7] L. A. Byers and C. M. Rudin , “Small Cell Lung Cancer: Where Do We Go From Here?,” Cancer 121, no. 5 (2015): 664–672. 25336398 10.1002/cncr.29098 PMC5497465

[8] A. Qin and G. P. Kalemkerian , “Treatment Options for Relapsed Small‐Cell Lung Cancer: What Progress Have We Made?,” Journal of Oncology Practice/American Society of Clinical Oncology 14, no. 6 (2018): 369–370. 10.1200/JOP.18.00278 29894661

[9] A. F. Farago and F. K. Keane , “Current Standards for Clinical Management of Small Cell Lung Cancer,” Translational Lung Cancer Research 7, no. 1 (2018): 69–79. 29535913 10.21037/tlcr.2018.01.16 PMC5835595

[10] V. Stephen , R. D. Liu , S. Shunichi , et al., “OA01.04 Five‐Year Survival in Patients With ES‐SCLC Treated With Atezolizumab in IMpower133: Imbrella a Extension Study Results,” Journal of Thoracic Oncology 18, no. 11 (2023): S44–S45.

[11] L. Paz‐Ares , M. Dvorkin , Y. Chen , et al., “Durvalumab Plus Platinum‐Etoposide Versus Platinum‐Etoposide in First‐Line Treatment of Extensive‐Stage Small‐Cell Lung Cancer (CASPIAN): A Randomised, Controlled, Open‐Label, Phase 3 Trial,” Lancet 394, no. 10212 (2019): 1929–1939. 31590988 10.1016/S0140-6736(19)32222-6

[12] L. Horn , A. S. Mansfield , A. Szczęsna , et al., “First‐Line Atezolizumab Plus Chemotherapy in Extensive‐Stage Small‐Cell Lung Cancer,” New England Journal of Medicine 379, no. 23 (2018): 2220–2229. 30280641 10.1056/NEJMoa1809064

[13] R. García‐Campelo , I. Sullivan , E. Arriola , et al., “SEOM‐GECP Clinical Guidelines for Diagnosis, Treatment and Follow‐Up of Small‐Cell Lung Cancer (SCLC) (2022),” Clinical & Translational Oncology 25, no. 9 (2023): 2679–2691. 37418123 10.1007/s12094-023-03216-3 PMC10425483

[14] Z. Zhang , Y. Li , Y. Dong , et al., “Successful Treatment of a Patient With Multiple‐Line Relapsed Extensive‐Stage Small‐Cell Lung Cancer Receiving Penpulimab Combined With Anlotinib: A Case Report,” Frontiers in Oncology 12 (2022): 846597. 35321433 10.3389/fonc.2022.846597 PMC8937034

[15] M. Das , S. K. Padda , J. Weiss , et al., “Advances in Treatment of Recurrent Small Cell Lung Cancer (SCLC): Insights for Optimizing Patient Outcomes From an Expert Roundtable Discussion,” Advances in Therapy 38, no. 11 (2021): 5431–5451. 34564806 10.1007/s12325-021-01909-1 PMC8475485

[16] M.‐J. Ahn , B. C. Cho , E. Felip , et al., “Tarlatamab for Patients With Previously Treated Small‐Cell Lung Cancer,” New England Journal of Medicine 389, no. 22 (2023): 2063–2075. 37861218 10.1056/NEJMoa2307980

[17] A. Dowlati , S. Babu , E. P. Hamilton , et al., “Abstract 1990MO—Sacituzumab Govitecan (SG) as Second‐Line (2L) Treatment for Extensive Stage Small Cell Lung Cancer (ES‐SCLC): Preliminary Results From the Phase II TROPiCS‐03 Basket Trial,” Annals of Oncology 34 (2023): S1062.

[18] Clinicaltrials.gov , “Study Comparing Tarlatamab With Standard of Care Chemotherapy in Relapsed Small Cell Lung Cancer (DeLLphi‐304),” 2024, https://classic.clinicaltrials.gov/ct2/show/NCT05740566.

[19] B. Besse , L. G. P.‐A. Peters , S. Peters , et al., “A Phase III Study of Lurbinectedin Alone or in Combination With Irinotecan vs. Investigator's Choice (Topotecan or Irinotecan) in Patients With Relapsed Small Cell Lung Cancer (SCLC; LAGOON Trial),” Journal of Clinical Oncology 41 (2023): TPS8613.

[20] E. Calvo , V. Moreno , M. Flynn , et al., “Antitumor Activity of Lurbinectedin (PM01183) and Doxorubicin in Relapsed Small‐Cell Lung Cancer: Results From a Phase I Study,” Annals of Oncology 28, no. 10 (2017): 2559–2566. 28961837 10.1093/annonc/mdx357 PMC5834091

[21] D. Morgensztern , B. Besse , L. Greillier , et al., “Efficacy and Safety of Rovalpituzumab Tesirine in Third‐Line and Beyond Patients With DLL3‐Expressing, Relapsed/Refractory Small‐Cell Lung Cancer: Results From the Phase II TRINITY Study,” Clinical Cancer Research 25, no. 23 (2019): 6958–6966. 31506387 10.1158/1078-0432.CCR-19-1133 PMC7105795

[22] D. R. Rivera , H. J. Henk , E. Garrett‐Mayer , et al., “The Friends of Cancer Research Real‐World Data Collaboration Pilot 2.0: Methodological Recommendations From Oncology Case Studies,” Clinical Pharmacology and Therapeutics 111 (2022): 283–292. 34664259 10.1002/cpt.2453 PMC9298732

[23] F. Franco , E. Carcereny , M. Guirado , et al., “Epidemiology, Treatment, and Survival in Small Cell Lung Cancer in Spain: Data From the Thoracic Tumor Registry,” PLoS One 16, no. 6 (2021): e0251761. 34077442 10.1371/journal.pone.0251761 PMC8171958

[24] M. E. Olmedo , M. Forster , V. Moreno , et al., “Efficacy and Safety of Lurbinectedin and Doxorubicin in Relapsed Small Cell Lung Cancer. Results From an Expansion Cohort of a Phase I Study,” Investigational New Drugs 39, no. 5 (2021): 1275–1283. 33704620 10.1007/s10637-020-01025-x PMC8426303

[25] C. C. Steffens , C. Elender , U. Hutzschenreuter , et al., “Treatment and Outcome of 432 Patients With Extensive‐Stage Small Cell Lung Cancer in First, Second and Third Line—Results From the Prospective German TLK Cohort Study,” Lung Cancer 130 (2019): 216–225. 30885347 10.1016/j.lungcan.2019.02.026

[26] N. Ready , A. F. Farago , F. de Braud , et al., “Third‐Line Nivolumab Monotherapy in Recurrent SCLC: CheckMate 032,” Journal of Thoracic Oncology 14, no. 2 (2019): 237–244. 30316010 10.1016/j.jtho.2018.10.003 PMC8050700

[27] Y. Xu , Z. Huang , H. Lu , et al., “Apatinib in Patients With Extensive‐Stage Small‐Cell Lung Cancer After Second‐Line or Third‐Line Chemotherapy: A Phase II, Single‐Arm, Multicentre, Prospective Study,” British Journal of Cancer 121, no. 8 (2019): 640–646. 31523058 10.1038/s41416-019-0583-6 PMC6889407

[28] A. D. Coutinho , M. Shah , O. E. Lunacsek , M. Eaddy , and J. P. Willey , “Real‐World Treatment Patterns and Outcomes of Patients With Small Cell Lung Cancer Progressing After 2 Lines of Therapy,” Lung Cancer 127 (2019): 53–58. 30642551 10.1016/j.lungcan.2018.11.009

[29] M. A. MustafaKaraagac , “Third‐Line Treatment is Associated With Prolonged Survival in Patients With Extensive‐Stage Small Cell Lung Cancer: A Single Center Experience,” 4, no. 2 (2020): 253–258.

[30] H. C. Chung , S. A. Piha‐Paul , J. Lopez‐Martin , et al., “Pembrolizumab After Two or More Lines of Previous Therapy in Patients With Recurrent or Metastatic SCLC: Results From the KEYNOTE‐028 and KEYNOTE‐158 Studies,” Journal of Thoracic Oncology 15, no. 4 (2020): 618–627. 31870883 10.1016/j.jtho.2019.12.109

[31] S. T. Keeping , S. Cope , K. Chan , et al., “Comparative Effectiveness of Nivolumab Versus Standard of Care for Third‐Line Patients With Small‐Cell Lung Cancer,” Journal of Comparative Effectiveness Research 9, no. 18 (2020): 1275–1284. 33140652 10.2217/cer-2020-0134

[32] C. Lv , X. Liu , Q. Zheng , et al., “Analysis of Topoisomerase I Expression and Identification of Predictive Markers for Efficacy of Topotecan Chemotherapy in Small Cell Lung Cancer,” Thoracic Cancer 9, no. 9 (2018): 1166–1173. 30058109 10.1111/1759-7714.12819 PMC6119620

[33] T. K. Owonikoko , M. Behera , Z. Chen , et al., “A Systematic Analysis of Efficacy of Second‐Line Chemotherapy in Sensitive and Refractory Small‐Cell Lung Cancer,” Journal of Thoracic Oncology 7, no. 5 (2012): 866–872. 22722788 10.1097/JTO.0b013e31824c7f4b PMC3381878

[34] P. F. Song , N. Xu , and Q. Li , “Efficacy and Safety of Anlotinib for Elderly Patients With Previously Treated Extensive‐Stage SCLC and the Prognostic Significance of Common Adverse Reactions,” Cancer Management and Research 12 (2020): 11133–11143. 33173346 10.2147/CMAR.S275624 PMC7646458

[35] E. Neeman , G. Gresham , N. Ovasapians , et al., “Comparing Physician and Nurse Eastern Cooperative Oncology Group Performance Status (ECOG‐PS) Ratings as Predictors of Clinical Outcomes in Patients With Cancer,” Oncologist 24, no. 12 (2019): e1460–e1466. 31227648 10.1634/theoncologist.2018-0882 PMC6975959

[36] D. E. O'Sullivan , W. Y. Cheung , I. A. Syed , et al., “Real‐World Treatment Patterns, Clinical Outcomes, and Health Care Resource Utilization in Extensive‐Stage Small Cell Lung Cancer in Canada,” Current Oncology 28, no. 4 (2021): 3091–3103. 34436036 10.3390/curroncol28040270 PMC8395392

[37] M. D. DiBonaventura , B. Shah‐Manek , K. Higginbottom , J. R. Penrod , and Y. Yuan , “Adherence to Recommended Clinical Guidelines in Extensive Disease Small‐Cell Lung Cancer Across the US, Europe, and Japan,” Therapeutics and Clinical Risk Management 15 (2019): 355–366. 30881001 10.2147/TCRM.S183216 PMC6400139

[38] P. Agapow , R. Mulla , N. Markuzon , L. H. Ottesen , and D. Meulendijks , “Systematic Review of Time to Subsequent Therapy as a Candidate Surrogate Endpoint in Advanced Solid Tumors,” Future Oncology 19, no. 23 (2023): 1627–1639. 37589145 10.2217/fon-2022-0616

[39] Clinicaltrials.gov , “A Study of Ifinatamab Deruxtecan Versus Treatment of Physician's Choice in Subjects With Relapsed Small Cell Lung Cancer,” (2024), https://classic.clinicaltrials.gov/ct2/show/NCT06203210.

