# Supplementary material for: Real‐World Response and Survival Outcomes and Treatment Patterns in Patients With Extensive Stage Small‐Cell Lung Cancer Receiving Third‐Line Treatment
Source: Cancer Rep (Hoboken). 2025 Aug 12;8(8):e70289. doi: 10.1002/cnr2.70289 (PMC12340977; doi:10.1002/cnr2.70289)
Supplement: Supplementary file 1 — Data S1. [file CNR2-8-e70289-s001.docx]

**Real-world response and survival outcomes and treatment patterns in patients with extensive stage small-cell lung cancer receiving third-line treatment**

Jair Bar, MD^1,2^, Qingqing Xu, PhD^3^, Sudeep Karve, PhD^3^, Pooja Hingorani, MD^3^, Amin A. Virani, PharmD^3^, Neal E. Ready, MD^4^

^1^ Institute of Oncology, Sheba Medical Center, Ramat Gan, Israel

^2^ Faculty of Medicine, Tel Aviv University, Tel Aviv-Yafo, Israel

^3^ AbbVie Inc., North Chicago, IL, USA

^4^ Duke Cancer Institute, Duke University, Durham, NC, USA

**SUPPLEMENTAL MATERIALS**

**Supplemental Methods – Prespecified systemic therapies[1]**

| **Drug type** | **Drug name** | **Note** |
| --- | --- | --- |
| **Chemotherapy** | \| etoposide \| \| --- \| \| irinotecan \| \| topotecan \| \| lurbinectedin \| \| paclitaxel \| \| docetaxel \| \| temozolomide \| \| cyclophosphamide (in CAV) \| \| doxorubicin (in CAV) \| \| vincristine (in CAV) \| \| vinorelbine \| \| gemcitabine \| \| bendamustine \| | CAV: cyclophosphamide/doxorubicin/vincristine |
| **ICI** | \| atezolizumab \| \| --- \| \| durvalumab \| \| nivolumab \| \| pembrolizumab \| | Plus other ICIs not listed in NCCN V2 2022:  avelumab  cemiplimab  ipilimumab |
| **Platinum agents** | \| carboplatin \| \| --- \| \| cisplatin \| | Plus other platinum agents not listed in NCCN V2 2022:  oxaliplatin  nedaplatin  lobaplatin |

ICI, immune checkpoint inhibitors; NCCN, National Comprehensive Cancer Network Guidelines.

**Supplemental Figure 1.** Consort diagram: patient selection and attrition.


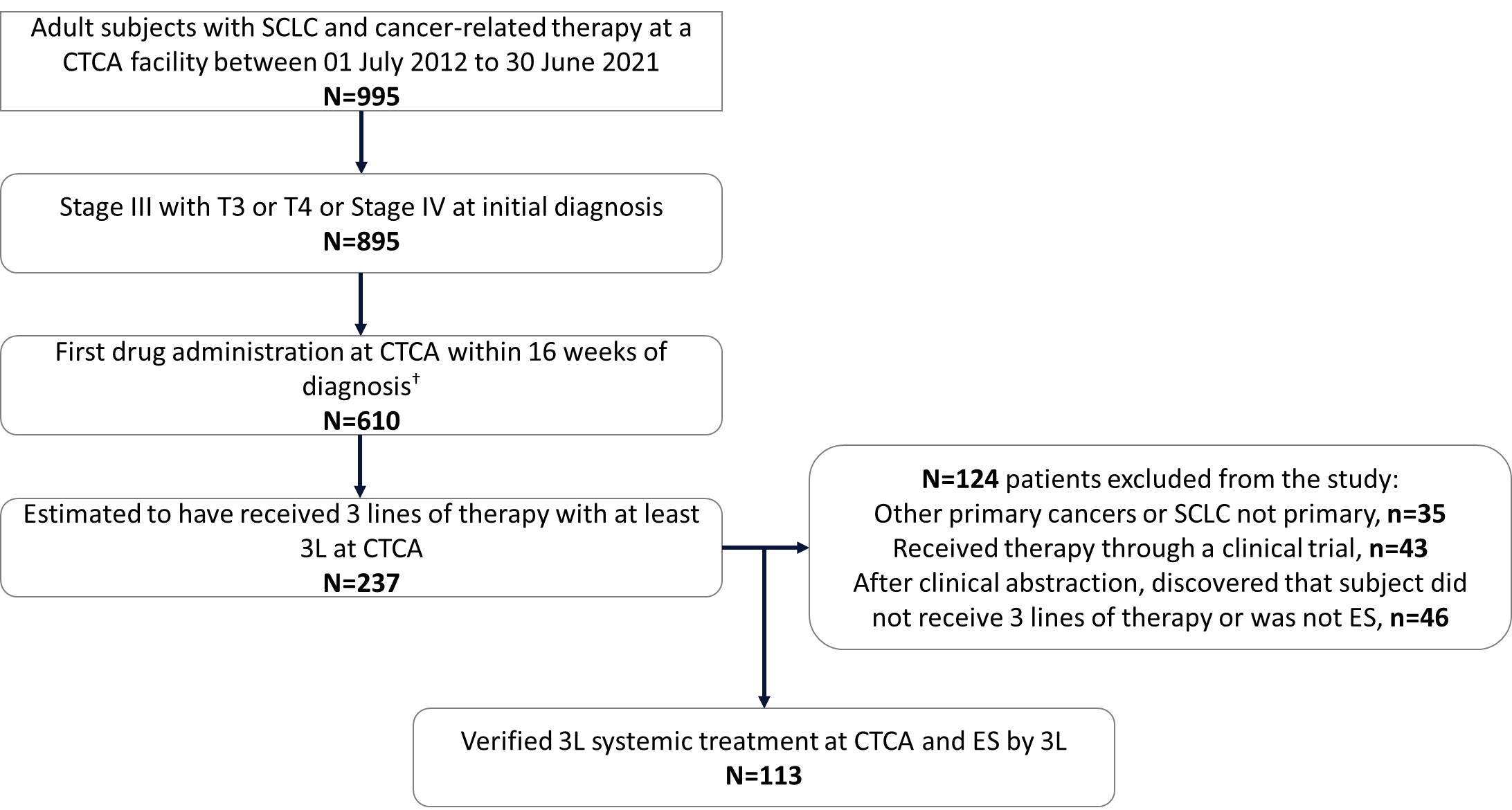


^†^This step was added to increase the likelihood of identifying patients who received 3L at CTCA.
Patients with a wide gap from diagnosis to CTCA care are more likely to have received therapy elsewhere before arriving to CTCA. 3L, third-line; CTCA, Cancer Treatment Centers of America; ES, extensive stage; SCLC, small-cell lung cancer.

**Supplemental Table 1. Treatment response in 1L and 2L among 3L patients**

| Response | 1L | 2L |
| --- | --- | --- |
|  | N (%) of 113 | N (%) of 113 |
| No response | 18 (15.9) | 62 (54.9) |
| Partial response | 85 (75.2) | 30 (26.5) |
| Complete response | 6 (5.3) | 3 (2.7) |
| Stable disease | 4 (3.5) | 18 (15.9) |

Note: these are physician reported observed values. 1L, first-line; 2L, second-line; 3L, third-line.

**References**

1. Referenced with permission from the NCCN Clinical Practice Guidelines in Oncology (NCCN Guidelines®) for **Small Cell Lung Cancer v.2.2022**. © National Comprehensive Cancer Network, Inc. **2022**. All rights reserved. Accessed **[December 14, 2024].** To view the most recent and complete version of the guideline, go online to NCCN.org. NCCN makes no warranties of any kind whatsoever regarding their content, use or application and disclaims any responsibility for their application or use in any way.
